# Novel Dual-Signal SiO_2_-COOH@MIPs Electrochemical Sensor for Highly Sensitive Detection of Chloramphenicol in Milk

**DOI:** 10.3390/s23031346

**Published:** 2023-01-25

**Authors:** Lingjun Geng, Mengyue Liu, Jingcheng Huang, Falan Li, Yanyan Zhang, Yemin Guo, Xia Sun

**Affiliations:** 1 School of Agricultural Engineering and Food Science, Shandong University of Technology, No. 266 Xincun Xilu, Zibo 255049, China; 2 Shandong Provincial Engineering Research Center of Vegetable Safety and Quality Traceability, No. 266 Xincun Xilu, Zibo 255049, China; 3 Zibo City Key Laboratory of Agricultural Product Safety Traceability, No. 266 Xincun Xilu, Zibo 255049, China

**Keywords:** dual-signal electrochemical sensor, surface molecularly imprinted polymers, NiFe Prussian blue analogue, SnS_2_ nanoflowers, chloramphenicol

## Abstract

In view of the great threat of chloramphenicol (CAP) to human health and the fact that a few producers have illegally used CAP in the food production process to seek economic benefits in disregard of laws and regulations and consumer health, we urgently need a detection method with convenient operation, rapid response, and high sensitivity capabilities to detect CAP in food to ensure people’s health. Herein, a molecularly imprinted polymer (MIP) electrochemical sensor based on a dual-signal strategy was designed for the highly sensitive analysis of CAP in milk. The NiFe Prussian blue analog (NiFe-PBA) and SnS_2_ nanoflowers were modified successively on the electrode surface to obtain dual signals from [Fe(CN)_6_]^3−/4−^ at 0.2 V and NiFe-PBA at 0.5 V. SiO_2_-COOH@MIPs that could specifically recognize CAP were synthesized via thermal polymerization using carboxylated silica microspheres (SiO_2_-COOH) as carriers. When the CAP was adsorbed by SiO_2_-COOH@MIPs, the above two oxidation peak currents decreased at the same time, allowing the double-signal analysis. The SiO_2_-COOH@MIPs/SnS_2_/NiFe-PBA/GCE sensor used for determining CAP was successfully prepared. The sensor utilized the interactions of various nanomaterials to achieve high-sensitivity dual-signal detection, which had certain innovative significance. At the same time, the MIPs were synthesized using a surface molecular imprinting technology, which could omit the time of polymerization and elution and met the requirements for rapid detection. After optimizing the experimental conditions, the detection range of the sensor was 10^−8^ g/L–10^−2^ g/L and the limit of detection reached 3.3 × 10^−9^ g/L (S/N = 3). The sensor had satisfactory specificity, reproducibility, and stability, and was successfully applied to the detection of real milk samples.

## 1. Introduction

Chloramphenicol (CAP) is a broad-spectrum antibiotic commonly used to treat bacterial infections caused by Gram-positive and Gram-negative bacteria [1]. Unfortunately, CAP ultimately enters and causes serious harm to the body [2,3]. In view of the huge threat of CAP to human health, many countries such as the United States, China, and Canada have issued decrees to explicitly prohibit the use of CAP in the food production process [4]. It is worrying that there are still many producers who pursue economic benefits by illegally using CAP in the food production process, regardless of the regulations and the health of consumers. Therefore, a detection method with convenient operation, rapid response, and high sensitivity capabilities is needed to detect CAP in food to preserve people’s health.

Among the various detection methods, electrochemical methods have received extensive attention due to their fast detection speeds, ease of operation, and low detection costs [5,6,7]. For the sake of achieving high specificity in detection, some recognition elements with specific recognition capabilities such as antibodies [8,9], aptamers [10,11], enzymes [12], and molecularly imprinted polymers (MIPs) [13,14,15] have been extensively utilized. Among the many recognition elements, MIPs are favored by many researchers because of their cost-effectiveness and high stability. MIPs are a class of polymers synthesized with target molecules as templates, which have a specific selective recognition function [16,17]. However, as most MIPs cannot achieve the purpose of the high-sensitivity detection of target molecules due to their poor electrical conductivity [18], the selection of nanomaterials with excellent conductivity to enhance the electrochemical signal is a problem that must be considered [19,20,21].

Prussian blue analog (PBA) is a novel compound formed by replacing the iron element in Prussian blue (PB) with other elements such as cobalt and nickel without destroying the crystal structure [22]. It has been widely utilized in electrochemical energy storage [23], electrocatalysis [24], and other fields. It is worth noting that the redox of multiple metal ions occurs in PBA, which means that under certain conditions, PBA itself can provide a detection signal for electrochemical analyses. As a transition metal chalcogenide [25], SnS_2_ has received extensive attention in the fields of photocatalysis [26] and gas sensing [27], on account of its two-dimensional layered structure, excellent chemical stability, and large surface area. At the same time, due to its good electrical conductivity and sandwich-like structure, which can provide reaction sites, SnS_2_ also has good application prospects in the field of electrochemistry. Controlling the morphology of MIPs in the synthesis process by selecting different carriers can make the particle size distribution more uniform. Compared with most other sensors using electropolymerization to prepare molecularly imprinted polymers, the electropolymerization and elution times were reduced, which was more in line with the requirements for rapid detection.

Here, we prepared a new dual-signal electrochemical sensor based on SiO_2_-COOH@MIPs for the quantitative detection and analysis of CAP in milk. The NiFe-PBA was initially modified on the electrode surface to furnish a second detection signal. The Ni ions in NiFe-PBA were oxidized under the action of SnS_2_ nanoflowers, which led to further amplification of the second detection signal [28]. Meanwhile, the three-dimensional flower-like structure of the SnS_2_ nanoflowers could also increase the specific surface area of the electrode, which provided more attachment sites for the next modified materials. The surface molecular imprinting technology (SMIT) was used to synthesize MIPs with SiO_2_ microspheres as the carriers, which acted as the recognition elements, and we carried out the specific recognition and detection of CAP. When the sensor was inserted into the CAP solution, the oxidation peak current of [Fe(CN)_6_]^3−/4−^ (I_probe_) at 0.20 V and the oxidation peak current of NiFe-PBA (I_substrate_) at 0.50 V could be observed to decrease simultaneously. The changes could be attributed to the re-adsorption of CAP by the imprinted cavity, hindering the electron transfer rate. The sensor achieved the high-sensitivity detection of CAP by analyzing the changes in the I_D_ value (I_D_ = I_probe_ + I_substrate_) after the CAP was re-adsorbed.

## 2. Experimental Section

The following components are introduced in the Supplementary Materials, namely the instruments and medicines required for the experiment (Supplementary Materials 2.1); the preparation procedure for the NiFe-PBA (Supplementary Materials 2.2), SnS_2_ nanoflowers (Supplementary Materials 2.3), SiO_2_ (Supplementary Materials 2.4.1), SiO_2_-COOH (Supplementary Materials 2.4.2), SiO_2_-COOH@MIPs, and SiO_2_-COOH@NIPs (Supplementary Materials 2.4.3); the procedure for the adsorption experiment and the pretreatment of GCE (Supplementary Materials 2.5 and 2.6); the parameters of the electrochemical measurement(Supplementary Materials 2.7); and the processing procedure for the actual sample (Supplementary Materials 2.8).

### Preparation of SiO_2_-COOH@MIPs/SnS_2_/NiFe-PBA/GCE Sensor

**Scheme 1:**
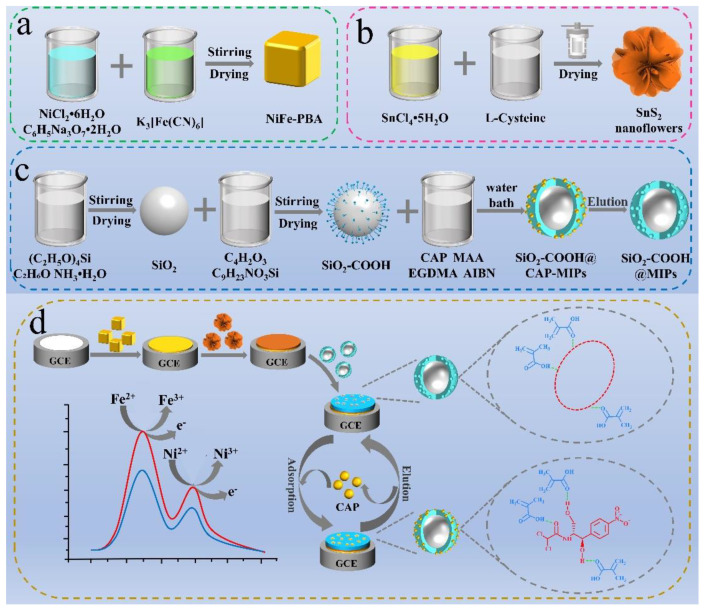
Preparation procedure for (**a**) NiFe-PBA, (**b**) SnS_2_ nanoflowers, and (**c**) SiO_2_-COOH@MIPs. (**d**) Schematic diagram of the novel dual-signal SiO_2_-COOH@MIPs electrochemical sensor preparation process.

We synthesized NiFe-PBA, SnS_2_, and SiO_2_-COOH@MIPs according to the steps in Scheme 1a–c (the specific synthesis process is described in detail in the Supplementary Materials). For the sake of obtaining a 1.0 mg/mL NiFe-PBA solution, the NiFe-PBA was dissolved in ultrapure water and sonicated for 15 min. Following the same operation steps, a 1.0 mg/mL SnS_2_ nanoflower solution was attained. Then, 10 μL of NiFe-PBA solution was dropped on the surface of the polished glassy carbon electrode to dry naturally at room temperature. After the NiFe-PBA solution was completely dry, 10 μL of SnS_2_ nanoflower solution was modified onto the NiFe-PBA/GCE surface. Ultimately, the sensor was obtained by modifying 10 μL of 10 mg/mL SiO_2_-COOH@MIPs on the SnS_2_/NiFe-PBA/GCE surface (Scheme 1d).

## 3. Results and Discussion

### 3.1. Characterization of the Prepared Materials

#### 3.1.1. NiFe-PBA

**Figure 1:**
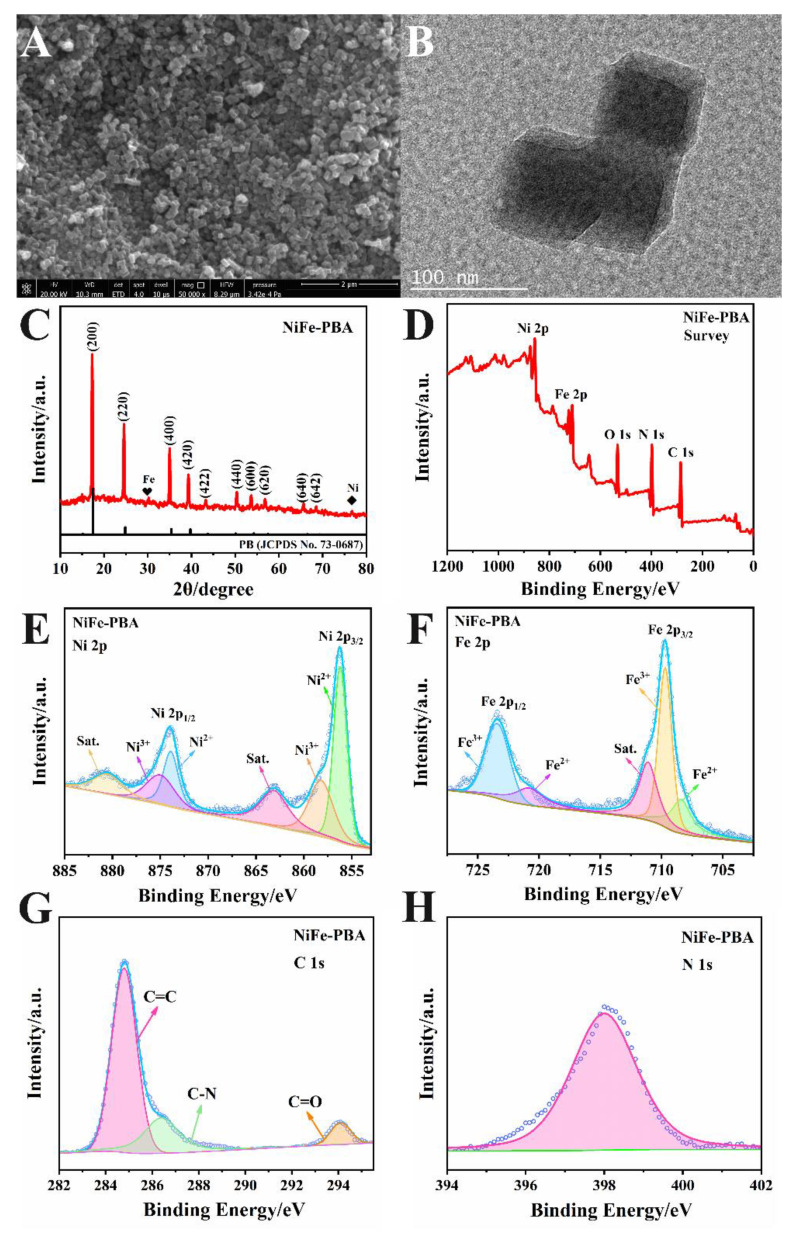
(**A**) SEM image, (**B**) TEM image, and (**C**) XRD image of NiFe-PBA. XPS spectra of NiFe-PBA: (**D**) full spectrum of XPS detection of NiFe-PBA; (**E**) Ni 2p spectrum, (**F**) Fe 2p spectrum, (**G**) C 1s spectrum, and (**H**) N 1s spectrum from the XPS detection of NiFe-PBA.

The morphological features of NiFe-PBA were characterized using scanning electron microscopy (SEM) and transmission electron microscopy (TEM). As shown in Figure 1A,B, the as-synthesized NiFe-PBA material had a typical nanocube structure with a smooth surface and uniform size, with an average size of 100 nm. By comparing the main diffraction peaks in the x-ray diffraction (XRD) detection results for NiFe-PBA with the data for PB (JCPDS No. 73-0687), we could more intuitively know that the NiFe-PBA had been successfully prepared and had high crystallinity (Figure 1C).

The ingredients in NiFe-PBA were further analyzed via X-ray photoelectron spectroscopy (XPS). The full measured spectrum of NiFe-PBA (Figure 1D) showed that there were five elements (Ni, Fe, C, N, and O) in the NiFe-PBA. Six peaks can be observed in the Figure 1E. The peaks of Ni^2+^ 2p_3/2_ and Ni^2+^ 2p_1/2_ appeared at 856.2 eV and 873.9 eV, respectively, and the two peaks at 858.3 eV and 875.1 eV belonged to Ni^3+^ 2p_3/2_ and Ni^3+^ 2p_1/2_, respectively. The remaining two peaks were noticeable satellite peaks (863.2 eV and 880.7 eV). In the spectrum of Fe 2p, five peaks could be observed (Figure 1F). The peaks of Fe^2+^ 2p_3/2_ and Fe^2+^ 2p_1/2_ appeared at 708.4 eV and 720.9 eV, respectively, and the two peaks at 709.7 eV and 723.4 eV belonged to Fe^3+^ 2p_3/2_ and Fe^3+^ 2p_1/2_, respectively. The remaining peak was a more obvious satellite peak (711.1 eV), which was consistent with previous reports in the literature [29]. The XPS spectra demonstrated that the two elements existed in NiFe-PBA in Ni^3+/2+^ and Fe^3+/2+^ valence states. The three peaks shown in the C 1s spectrum (Figure 1G) at 284.8, 286.4, and 294.1 eV correspond to C=C, C-N, and C=O, respectively. There was only one peak in the N 1s spectrum at 398 eV (Figure 1H).

#### 3.1.2. SnS_2_ Nanoflowers

In this study, SEM, TEM, mapping, XPS, XRD, and the Randles–Sevcik equation were used to fully characterize and analyze the properties of SnS_2_ nanoflowers. The relevant experimental data are shown in Supplementary Material 3.1.2.

#### 3.1.3. SiO_2_-COOH@MIPs

SEM, TEM, Fourier transform infrared (FT-IR) spectroscopy, and a thermo-gravimetric analysis (TGA) were used to characterize and analyze SiO_2_-COOH@MIPs. The relevant experimental data are shown in Supplementary Material 3.1.3.

### 3.2. Electrochemical Investigation of SiO_2_-COOH@MIPs/SnS_2_/NiFe-PBA/GCE

**Figure 2:**
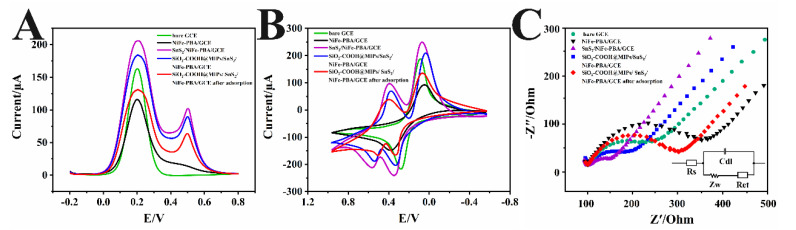
(**A**) DPV, (**B**) CV, and (**C**) EIS curves of electrode surfaces modified with different materials. Electrochemical measurements were performed in solutions containing 5 mmol/L[Fe(CN)_6_]^3−/4−^ and 0.1 mol/L KCl.

The electrochemical performances of electrodes modified with different materials were characterized using the differential pulse voltammetry (DPV) technique. As shown in Figure 2A, only one oxidation peak appeared in the DPV curve of the bare electrode. Dropping the poorly conductive NiFe-PBA onto the bare electrode surface resulted in a decrease in the oxidation peak current at 0.20 V and the appearance of a new oxidation peak at 0.50 V. However, the peak value at 0.50 V was too minute to achieve the purpose of detection. Therefore, we introduced SnS_2_ nanoflowers with good electrical conductivity, and the three-dimensional flower-like structure of the SnS_2_ nanoflowers increased the specific surface area and provided more attachment sites. Notably, when SnS_2_ nanoflowers were decorated on the NiFe-PBA/GCE surface, the peak current of the oxidation peaks at 0.20 V and 0.50 V was significantly enhanced and reached a maximum value. Since the as-prepared SiO_2_-COOH @MIPs did not have electrical conductivity, the oxidization peak-to-peak current at [Fe(CN)_6_]^3−/4−^ and NiFe-PBA were both weakened when the SiO_2_-COOH@MIPs were decorated on the SnS_2_/NiFe-PBA/GCE surface. However, the surfaces of SiO_2_-COOH@MIPs had abundant imprinted cavities similar in structure and size to the template molecule after elution, which provided channels for electron transfer. The two peak currents were less weakened. When the as-prepared electrode was placed in a certain concentration of CAP solution, the CAP was adsorbed specifically to fill the imprinted cavities on the SiO_2_-COOH@MIPs, which hindered the electron transfer. The peak currents of the two oxidation peaks were significantly reduced. The SiO_2_-COOH@MIPs/SnS_2_/NiFe-PBA/GCE sensor had been successfully fabricated as demonstrated by the above tests. Combined with Figure 2B, this allowed us to better prove the above conclusion.

The different assembly steps of the sensor were further characterized using EIS, and the results are shown in Figure 2C. Compared with the bare electrode, when the NiFe-PBA with poor conductivity was modified on the electrode surface, the impedance increased and the diameter of the semicircle became larger. When SnS_2_ with good conductivity was further modified on the electrode surface, the impedance decreased and the diameter of the semicircle became smaller. When the electrode surface was further modified with SiO_2_-COOH@MIPs, the impedance increased due to the poor conductivity of SiO_2_-COOH@MIPs. As the adsorption process went on, the imprinted cavity on the electrode surface was occupied, which hindered the transfer of electrons and further increased the impedance. The results of the EIS analysis were similar to those of the CV and DPV analyses, which further proved that the sensor preparation process was successful.

### 3.3. Optimization of Conditions

Here, we define the ΔI_D_ value (ΔI_D_ = ΔI_probe_ + ΔI_substrate_) that represents the sum of the current differences between elution and incubation.

#### 3.3.1. Molar Ratio of CAP to MAA

**Figure 3:**
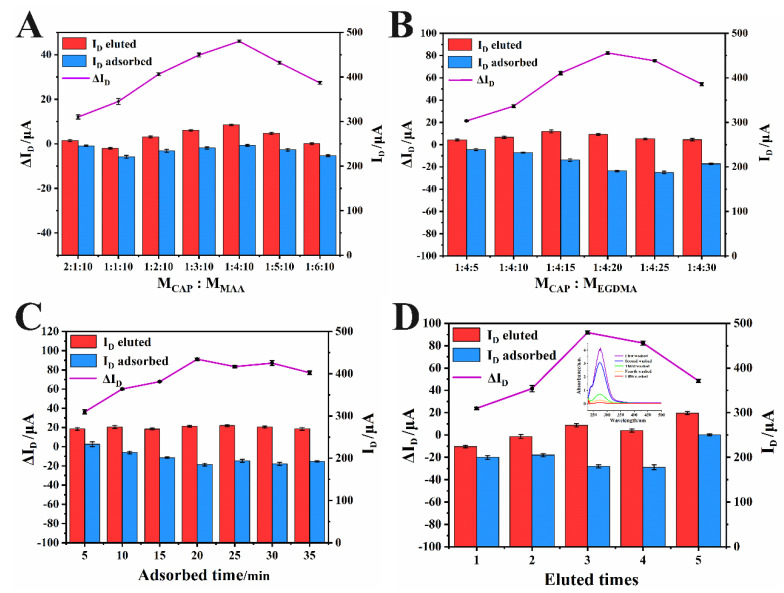
Discovering the best experimental parameters: (**A**) M_CAP_/M_MAA_ ratio; (**B**) M_CAP_/M_EGDMA_ ratio; (**C**) adsorption time; (**D**) elution times (illustration: the UV absorption spectrum of CAP in eluate after elution times of t = 1, 2, 3, 4 and 5; *n* = 3).

The ability of MIPs to re-adsorb CAP varied with the molar ratio of CAP to MAA (Figure 3A). When the M_CAP_/M_MAA_ ratio increased from 2:1 to 1:4, the ΔI_D_ value steadily increased. However, when the molar ratio of CAP to MAA increased from 1:4 to 1:6, the ΔI_D_ value gradually decreased. This phenomenon could be explained by the copolymerization of the crosslinker and functional monomer during the actual reaction. The presence of excessive functional monomers could lead to polymerization between the functional monomers and reduce the number of imprinted cavities with specific recognition ability, thereby reducing the adsorption capacity of the MIP. We could conclude that the optimal M_CAP_.M_MAA_ ratio was 1:4.

#### 3.3.2. Molar Ratio of CAP to EGDMA

Figure 3B shows the effect of the molar ratio of the template molecule CAP to the crosslinker EGDMA on the ability of MIPs to re-adsorb CAP. The ΔI_D_ value also changed significantly with the change in the amount of crosslinking agent added. The ΔI_D_ value continually increased when the M_CAP_:M_EGDMA_ ratio rose from 1:5 to 1:20. This was because the more EGDMA we added, the more CAP was bound when forming the MIPs.

It was common sense that the more EGDMA we added, the more the CAP formed MIPs and the more specific recognition cavities formed after elution. However, the ΔI_D_ values continued to decrease when the M_CAP_:M_EGDMA_ ratio rose from 1:20 to 1:30 because the excess crosslinker interfered with the ability of the MIPs to specifically recognize CAP. By contrasting the ΔI_D_ value, the optimal molar ratio of CAP, MAA, and EGDMA was determined to be 1:4:20.

#### 3.3.3. Adsorption Time

The performance of the sensor was also affected by the adsorption time. As the adsorption time steadily increased, the ΔI_D_ value also increased gradually, which could be attributed to the fact that a large number of target molecules were specifically adsorbed into the cavity of the MIPs with the increase in time, hindering the transfer of electrons. The maximum value of ΔI_D_ was reached at 20 min (Figure 3C). When the adsorption time continued to extend, we found that the ΔI_D_ value decreased slightly. This phenomenon could be attributed to the expansion and deformation of MIPs caused by prolonged immersion, resulting in the detachment of some target molecules from the MIPs.

#### 3.3.4. Elution Times

The elution times were also one of parameters that needed to be optimized for the sensor because the number of imprinted cavities with specific recognition target molecules was directly affected by the elution times. According to the UV absorption spectrum of the eluate in Figure 3D (inset), the UV absorption peak (278 nm) of CAP in the fifth eluate was close to zero, indicating that the template molecule CAP could be completely removed after five elution phases. However, combined with the statistical graph in Figure 3D, the ΔI_D_ reached the maximum value in the third elution phase. When the elution continued, the ΔI_D_ showed a significant downward trend. This might have been because the excessive number of elution phases reduced the imprinted cavity, which could specifically recognize the target molecule, by disrupting the 3D imprinted structure of the MIPs. Therefore, three elution phases was selected as the optimal number.

### 3.4. Adsorption Studies

**Figure 4:**
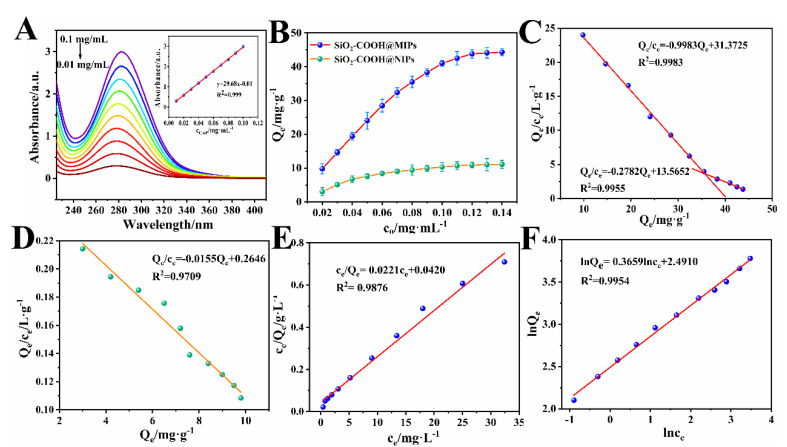
(**A**) UV absorbance curves of CAP solutions with different concentrations (0.01 mg/mL–0.1 mg/mL) (illustration: the standard curve obtained by fitting different CAP concentrations with their corresponding absorbance values). (**B**) Adsorption isotherms for the adsorption of different concentrations of CAP by SiO_2_-COOH@MIPs and SiO_2_-COOH@NIPs. Scatchard model fitting curves of adsorption isotherms of SiO_2_-COOH@MIPs (**C**) and SiO_2_-COOH@NIPs (**D**). The adsorption isotherm fitting curves of SiO_2_-COOH@MIPs (Langmuir adsorption model (**E**); Freundlich adsorption model (**F**)) (*n* = 3).

The absorbance values of different concentrations (0.01 mg/mL–0.1 mg/mL) of CAP standard solutions were measured using a UV spectrophotometer, and the results are shown in Figure 4A. Then, taking the concentration of the CAP solution as the abscissa and the absorbance value as the ordinate, the standard curve was drawn (R^2^ = 0.999), as shown in the illustration in Figure 4A.

#### 3.4.1. Adsorption Isotherm Analysis

The adsorption isotherm curve was one of the important factors for studying the adsorption mechanism, which could reflect the interactions between the template molecules and SiO_2_-COOH@MIPs during the adsorption process [30]. The equilibrium adsorption capacity (Q_e_) of SiO_2_-COOH@MIPs was 44.3 mg/g, which was 3.6 times higher than that of SiO_2_-COOH@NIPs (12.1 mg/g) (Figure 4B). Compared with SiO_2_-COOH@NIPs, the high specific binding ability of SiO_2_-COOH@MIPs to CAP could be attributed to the large number of imprinted cavities in the SiO_2_-COOH@MIPs, which could specifically recognize CAP.

The Scatchard equation is a commonly used analytical method to analyze the binding properties of MIPs. The Scatchard equation formula is as follows [31]:

(1) $$\frac{Q_{e}}{C_{e}}=-\frac{Q_{m}{-Q}_{e}}{K_{d}}$$

where *Q_m_* (mg/g) is the maximum adsorption capacity of the binding site and *K_d_* (mg/L) is the dissociation constant of the binding site.

By performing a Scatchard model analysis on the adsorption isotherm, the fitting curve as shown in Figure 4C,D could be obtained, and the data obtained via fitting and calculation are recorded in Table S1. From Figure 4C, we found that there were two fitting curves with different slopes in the Scatchard model analysis of the CAP adsorption by SiO_2_-COOH@MIPs, indicating that there might be two different binding sites for the adsorption of CAP by SiO_2_-COOH@MIPs. A further analysis showed that the *K_d_* values of the two fitted curves were 1.286 mg/L and 3.595 mg/L, respectively. It could be concluded that the two binding sites exhibited high affinity and low affinity, respectively. Comparing the *K_d_* value of the SiO_2_-COOH@NIPs with those of the SiO_2_-COOH@MIPs, we were surprised to find that the *K_d_* value of the SiO_2_-COOH@NIPs was much larger than the two *K_d_* values of the SiO_2_-COOH@MIPs. This phenomenon occurred because the SiO_2_-COOH@NIPs did not specifically recognize and bind to the imprinted cavity of CAP.

The adsorption behavior of the SiO_2_-COOH@MIPs was further analyzed using Langmuir and Freundlich isothermal models (Figure 4E,F), where the Langmuir model was suitable for monolayer adsorption and the Freundlich model was suitable for multilayer adsorption. The data obtained from the fitting calculation are recorded in Table S2. The Langmuir formula and the Freundlich formula are as follows [32]:

(2) $$\frac{C_{e}}{Q_{e}}=\frac{C_{e}}{Q_{m}}+\frac{1}{K_{L}{\cdot Q}_{m}}$$

(3) $${\ln Q}_{e}{=\ln K}_{F}+\frac{{\ln C}_{e}}{n}$$

where *K_L_* (L/mg) is the adsorption constant of the Langmuir formula, *K_F_* (L/mg) is the adsorption constant of the Freundlich formula, and *n* is the constant of the adsorption strength of the polymer. According to Table S2, the value of 1/n was 0.366 (0.1 < 1/*n* < 1) [33]. This indicated that the SiO_2_-COOH@MIPs had good adsorption capacity for CAP under the optimal experimental conditions. At the same time, the table shows that the correlation value of the Langmuir model (R^2^ = 0.988) was smaller than that of the Freundlich model (R^2^ = 0.994), which indicated that the process of CAP adsorption was more suitably described by the Freundlich model, while the adsorption process was dominated by multi-molecular layer adsorption.

#### 3.4.2. Adsorption Kinetics Analysis and Selective Adsorption Analysis

In this study, the adsorption kinetics and selective adsorption of SiO_2_-COOH@MIPs and SiO_2_-COOH@NIPs were investigated. The pseudo-first-order kinetics equation and pseudo-second-order kinetics equation were used to fit the adsorption data. The specific data are recorded in Supplementary Material 3.4.2.

### 3.5. Capability Assessment of the SiO_2_-COOH@MIPs/SnS_2_/NiFe-PBA/GCE Sensor

**Figure 5:**
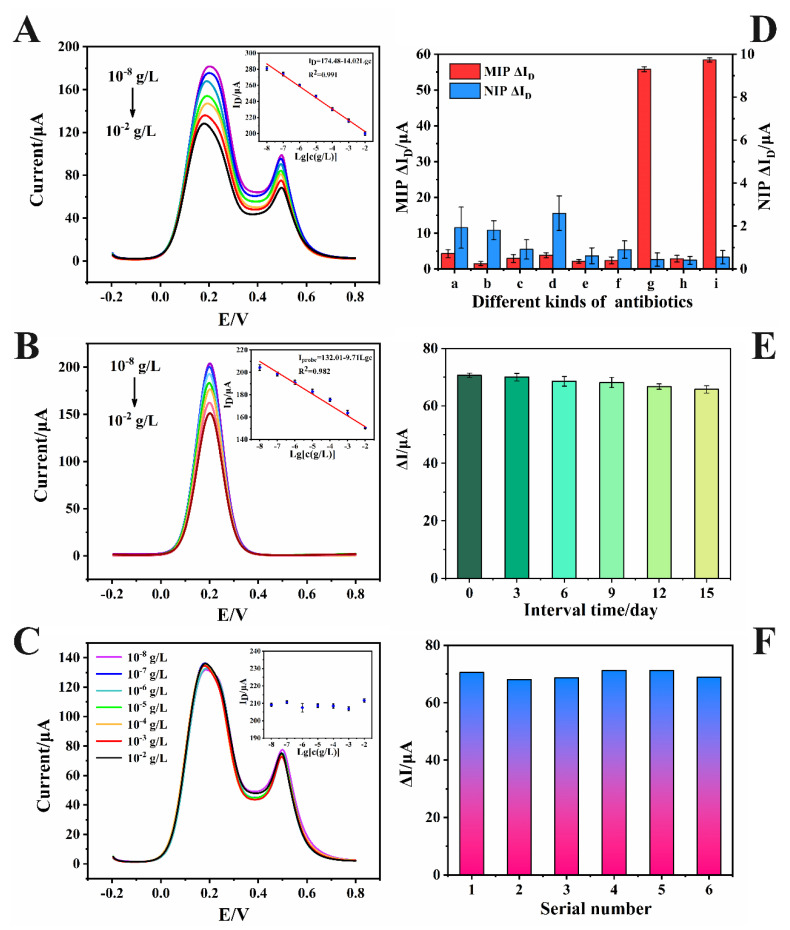
(**A**) DPV response for SiO_2_-COOH@MIPs/SnS_2_/NiFe-PBA/GCE in solutions containing 5 mmol/L [Fe(CN)_6_]^3−/4−^ and 0.1 mol/L KCl relative to various concentrations of CAP from 10^−8^ g/L to 10^−2^ g/L. Illustration: Plot of linear calibration with R^2^ = 0.991. (**B**) DPV response for SiO_2_-COOH@MIPs/SnS_2_/GCE in solutions containing 5 mmol/L [Fe(CN)_6_]^3−/4−^ and 0.1 mol/L KCl relative to various concentrations of CAP from 10^−8^ g/L to 10^−2^ g/L. Illustration: Plot of linear calibration with R^2^ = 0.982. (**C**) DPV response for SiO_2_-COOH@NIPs/SnS_2_/NiFe-PBA/GCE in solutions containing 5 mmol/L [Fe(CN)_6_]^3−/4−^ and 0.1 mol/L KCl relative to various concentrations of CAP from 10^−8^ g/L to 10^−2^ g/L. Illustration: There was no linear relationship. The SiO_2_-COOH@MIPs/SnS_2_/NiFe-PBA/GCE plots of (**D**) specificity ((a) florfenicol; (b) ofloxacin; (c) norfloxacin; (d) thiamphenicol; (e) kanamycin; (f) tetracycline; (g) chloramphenicol; (h) a–f; (i) a–g.), (**E**) stability, and (**F**) reproducibility (*n* = 3).

Under the optimum tentative conditions, the sensor was used to detect CAP solutions with different concentrations (10^−8^ g/L-10^−2^ g/L) to test the capacity of the sensor to detect and analyze CAP. When the CAP concentration gradually increased, the oxidization peak-to-peak current of the [Fe(CN)_6_]^3−/4−^ and NiFe-PBA gradually decreased; that is, the I_D_ value gradually decreased (Figure 5A). There was a good linear relationship between the obtained I_D_ value and the logarithm of the CAP concentration. The linear equation was I_D_ = 174.48 − 14.02 Lgc (R^2^ = 0.991), and the LOD was 3.3 × 10^−9^ g/L (S/N = 3).

To demonstrate the merits of dual-signal MIPs sensors, we also prepared single–signal SiO_2_-COOH@MIPs/SnS_2_/GCE sensors (Figure 5B) as well as SiO_2_-COOH@NIPs/SnS_2_/NiFe-PBA/GCE sensors (Figure 5C) as controls. It can be seen from Figure 5B that the detection range of the single–signal sensor was 10^−8^ g/L-10^−2^ g/L, and the linear equation was I_probe_ = 132.01 − 9.71 Lgc (R^2^ = 0.982). By comparing the sensitivity of the single–signal sensor (9.71 μA/(g/L)) and the dual-signal sensor (14.02 μA/(g/L)), the sensitivity of the dual-signal sensor was better, and higher sensitivity detection could be achieved. It can be seen from Figure 5C that after the sensor modified by SiO_2_-COOH@NIPs was adsorbed in distinct concentrations of CAP solution, no obvious linear relationship between the I_D_ value and CAP concentration was found. Table 1 summarizes some of the published methods used for CAP detection. From the comparison data, it was obvious that the sensor designed in this paper had better detection ability.

To explore the specificity of the SiO_2_-COOH@MIPs/SnS_2_/NiFe-PBA/GCE sensor and the SiO_2_-COOH@NIPs/SnS_2_/NiFe-PBA/GCE sensor, this study measured the ΔI_D_ value in a mixed solution containing 10^−4^ g/L CAP and 10^−3^ g/L of interfering substances. As shown in Figure 5D, the ability of the SiO_2_-COOH@MIPs to specifically recognize the CAP was not affected by the interfering substances, and the ability of the SiO_2_-COOH@MIPs to recognize the CAP was much higher than the SiO_2_-COOH@NIPs.

For the purpose of testing the stability of the sensor (Figure 5E), we prepared 24 SiO_2_-COOH@MIPs/SnS_2_/NiFe-PBA/GCE electrodes under optimal and identical conditions, divided them into 6 groups, and stored them at 4 °C in a refrigerator. We removed a group of electrodes every three days, put them into the CAP solution at a concentration of 1.0 × 10^−3^ g/L for adsorption, and calculated the ΔI_D_ value. After 15 days, the ΔI_D_ value of the last set of electrodes remained at 93.91% of the initial value. The above series of experiments fully demonstrated the excellent stability of the sensor. The reproducibility test was carried out using 6 electrodes (Figure 5F). The sensor prepared under optimal conditions was immediately placed in a CAP solution at a concentration of 10^−3^ g/L for adsorption and the ΔI_D_ value was calculated. The relative standard deviation (RSD) of the ΔI_D_ value of the 6 GCEs was merely 1.85%, indicating that the sensor had excellent reproducibility.

### 3.6. Real Sample Analysis

We tested the performance of the constructed sensor in the detection of real samples using the standard additive method. Table 2 shows the detection results of the sensor in the actual sample. The recovery range of the standard addition was 97.7~104.1%, and the RSD was less than 4.68%. The detection results showed that the SiO_2_-COOH@MIPs/SnS_2_/NiFe-PBA/GCE sensor had gratifying detection accuracy and convincing reliability in real sample detection, and was a promising CAP detection tool.

## 4. Conclusions

In this paper, a molecularly imprinted electrochemical sensor based on a dual-signal analysis was constructed for the detection of chloramphenicol in milk. The prepared SiO_2_-COOH@MIPs/SnS_2_/NiFe-PBA/GCE using the combination of molecular imprinting technology and electrochemical sensing technology had a wider detection range (10^−8^ g/L–10^−2^ g/L), a lower LOD (3.3 × 10^−9^ g/L), and higher sensitivity (14.02 μA/(g/L)), with good specificity and reproducibility, satisfactory stability, and excellent recovery (97.7~104.1%) in actual sample detection at the same time. This paper was based on the interactions of multiple nanomaterials to achieve dual-signal high-sensitivity detection, which had certain innovative significance. At the same time, the detection method proposed in this paper also had certain reference significance for the detection of target substances in other fields. However, molecularly imprinted electrochemical sensors also face some challenges, such as the incomplete elution of template molecules and difficulties with single-synthesis methods. Therefore, exploring new elution methods and synthesis methods in future work is still an important research direction.

## Figures and Tables

**Table 1:** Comparative study of this work with previously reported sensors for the detection of CAP.

Method	Linear Range (ng/mL)	LOD (ng/mL)	Reference
ELISA	1.0 × 10^−2^–1.0 × 10^2^	9.0 × 10^−3^	[34]
HPLC	12.3–2.2 × 10^3^	0.14	[35]
HPLC-MSIMS	0.02–5	2.0 × 10^−2^	[36]
Colorimetry	8.5 × 10^−3^–4.1	8.5 × 10^−3^	[37]
Fluorescence	3.2 × 10^−2^–3.2	1.9 × 10^−2^	[38]
ECL-enzyme-Apt	3.2 × 10^−4^–0.32	3.2 × 10^−4^	[39]
Electrochemistry-MIPs	0.1–1.0 × 10^3^	3.3 × 10^−2^	[40]
Electrochemistry-MIPs	1.0 × 10^−2^–1.0 × 10^4^	3.3 × 10^−3^	This work

**Table 2:** Recovery rates and RSD with different concentrations of CAP in milk samples (*n* = 3).

Sample	Spiked (g/L)	Detected (g/L)	Recovery (%)	RSD (%)
Milk	0	0	-	-
	1.0 × 10^−7^	0.981 × 10^−7^	98.1	2.19
	1.0 × 10^−6^	0.977 × 10^−6^	97.7	2.73
	1.0 × 10^−5^	1.041 × 10^−5^	104.1	3.44
	1.0 × 10^−4^	0.992 × 10^−4^	99.2	4.68
	1.0 × 10^−3^	1.027 × 10^−3^	102.7	4.31

## Data Availability

Not applicable.

## Supplementary Materials

The following supporting information can be downloaded at: https://www.mdpi.com/article/10.3390/s23031346/s1, Figure S1. (A) SEM image of NiFe-PBA SnS_2_ nanoflowers. (B) TEM image of SnS_2_ nanoflowers. (C) EDS mapping image of SnS_2_ nanoflowers. (D) EDS spectrum of SnS_2_ nanoflowers. (E) XRD image of SnS_2_ nanoflowers. XPS spectra of SnS_2_ nanoflowers: (F) full-survey spectrum, (G) S 2p spectrum and (H) Sn 3d spectrum. Figure S2. Cyclic voltammetry curves of GCE (A) and SnS_2_/GCE (C) at different scan rates. Linear relationship of the square root of scan rate to oxidation peak current and reduction peak current, respectively (GCE(B); SnS_2_/GCE (D)). Figure S3. (A) SEM image of SiO_2_; (B) SEM image of SiO_2_-COOH@MIPs; (C) TEM image of SiO_2_; (D) TEM image of SiO_2_-COOH@MIPs; (E) FTIR images and (F) TGA images of (a) SiO_2_; (b) SiO_2_-COOH and (c) SiO_2_-COOH@MIPs. Figure S4. (A) Adsorption kinetics curves for the adsorption of different concentrations of CAP by SiO_2_-COOH@MIPs and SiO_2_-COOH@NIPs. Pseudo-first-order kinetics equation (B), pseudo-second-order kinetics equation (C) of SiO_2_-COOH@MIPs in the adsorption kinetics test of CAP. (D) Chemical structures of five antibiotics CAP, FFC, OFX, NOR and TAP. (E) Adsorption capacity of SiO_2_-COOH@MIPs and SiO_2_-COOH@NIPs for CAP, FFC, OFX, NOR and TAP. Table S1: Scatchard parameters of SiO_2_-COOH@MIPs and SiO_2_-COOH@NIPs for the adsorption of CAP. Table S2: Langmuir and Freundlich parameters of SiO_2_-COOH@MIPs and SiO_2_-COOH@NIPs for the adsorption of CAP. Table S3: Pseudo-first-order and Pseudo-second-order parameters of SiO_2_-COOH @MIPs for the adsorption of CAP [27,28,30,41,42,43,44,45,46].
