# Supplementary material for: Novel Dual-Signal SiO2-COOH@MIPs Electrochemical Sensor for Highly Sensitive Detection of Chloramphenicol in Milk
Source: Sensors (Basel). 2023 Jan 25;23(3):1346. doi: 10.3390/s23031346 (PMC9920509; doi:10.3390/s23031346)
Supplement: Supplementary file 1 [file sensors-23-01346-s001.zip › supplementary materials.pdf]

Novel Dual-Signal SiO_2-COOH@MIPs Electrochemical Sensor for Highly Sensitive Detection of Chloramphenicol in Milk

Lingjun Geng[a,b,c], Mengyue Liu[a,b,c], Jingcheng Huang[a,b,c], Falan Li[a,b,c], Yanyan Zhang [a,b,c], Yemin Guo [a,b,c], Xia Sun[a,b,c,*]

[a]School of Agricultural Engineering and Food Science, Shandong University of Technology, No. 266 Xincun Xilu, Zibo, Shandong, 255049, China

[b]Shandong Provincial Engineering Research Center of Vegetable Safety and Quality Traceability, No. 266 Xincun Xilu, Zibo, Shandong, 255049, China

[c]Zibo City Key Laboratory of Agricultural Product Safety Traceability, No. 266 Xincun Xilu, Zibo, Shandong, 255049, China

*Corresponding author: Professor Xia Sun (X. Sun).

E-mail addresses: sunxia2151@sina.com (X. Sun).

2. Experimental section

2.1 Apparatus and reagents

The electrochemical workstation (CHI-660D) used in the DPV method was purchased from Shanghai Chenhua Instrument Co., Ltd. The hydrothermal reactor (HTG) was purchased from Anhui Kemi Instrument Co., Ltd. UV spectrophotometer (UV-2550) was obtained from Shimadzu Instruments Co., Ltd. Fourier transform infrared spectrometer (Nicolet 5700) was purchased from Thermoelectric Nicoli Instruments Inc. The US Environmental Scanning Electron Microscope (quantum-250) was responsible for taking SEM images of NiFe-PBA, SnS_2 nanoflowers and SiO_2-COOH@MIPs. American transmission electron microscope (WJGS-032) was responsible for collecting TEM images of NiFe-PBA, SnS_2 nanoflowers and SiO_2-COOH@MIPs. The X-ray photoelectron spectroscopy (XPS) of NiFe-PBA, and SnS_2 nanoflowers were collected by X-ray photoelectron spectroscopy (PHI Quan-tera II). The X-ray diffraction patterns (XRD) of NiFe-PBA, and SnS_2 nanoflowers were collected by X-ray diffractometer (D8 ADVANCE). Thermogravimetric analysis (TGA) data was measured by a comprehensive thermal analyzer (SDT650).

Antibiotics (chloramphenicol, florfenicol, ofloxacin, norfloxacin, thiamphenicol, kanamycin, tetracycline), $NiCl_2 \cdot 6H_2O$, $SnCl_4 \cdot 5H_2O$, ethylene glycol methacrylate (EGDMA), Maleic anhydride and 3-aminopropyltriethoxysilane (APTES) were bought from Shanghai Aladdin Reagent Co., Ltd. Methacrylic acid (MAA), acetonitrile, absolute ethanol, KCl, glacial acetic acid, methanol, N,N-dimethylformamide (DMF), ethyl orthosilicate (TEOS), a mmonium hydroxide and methacrylic acid (MAA) were bought from Sinopharm Chemical Reagent (Shanghai) Co., Ltd.

Azobisisobutyronitrile (AIBN) was purchased from Shanghai Xianding Biotechnology Co., Ltd. Buy Potassium Ferricyanide and Potassium Ferrocyanide from Shanghai Bodi Chemical Co., Ltd.

2.2 Preparation of NiFe-PBA

NiFe-PBA was prepared according to previous reports[1]. Briefly, 1.0 mmol $K_3[Fe(CN)_6]$ was dissolved in 40 mL ultrapure water to form solution A. Then 1.0 mmol $NiCl_2 \cdot 6H_2O$ and 2.0 mmol sodium citrate dihydrate were dissolved in 40 mL ultrapure water, so solution B was formed. Mix the prepared solution A and solution B and stir for 24 h at room temperature. During stirring, the mixture was observed to gradually change from clear to cloudy. Then, the reacted solution was centrifuged by high-speed centrifuge to gather the precipitate, and the precipitate was washed with ultrapure water for several times by centrifugation. After completing the above operations, the washed precipitate was dried in an oven at 60 °C for 12 h, and the obtained brown powder was NiFe-PBA.

2.3 Preparation of SnS_2 nanoflowers

According to the following steps, SnS_2 nanoflowers were synthesized. In short, 0.35 g $SnCl_4 \cdot 5H_2O$ was added to 60 mL ultrapure water containing 0.25 g L-cysteine, sonicated until completely dissolved. And the mixture was gently stirred at room temperature for 15 min. Subsequently, the mixed solution was transferred to hydrothermal reactor and reacted in a thermostatic electronic oven at 160 °C for 24 h. Wait for the temperature of the autoclave to drop to room temperature by natural cooling, and then wash the precipitate collected by centrifugation several times with ultrapure water and absolute ethanol. Almost immediately, the washed precipitate was dried in a vacuum oven at 60 °C for 12 h, and the obtained dark brown powder was SnS_2 nanoflowers.

2.4 Preparation of MIPs and NIPs

2.4.1 Synthesis of SiO_2 microspheres

First, solution A containing 1.5 mL of TEOS and 3.0 mL of absolute ethanol and solution B containing 4 mL of $NH_3 \cdot H_2O$ and 47 mL of absolute ethanol were placed in a constant temperature magnetic stirring water bath (32°C/30 min). Then, the mixture obtained by adding solution A to solution B at a constant rate was placed in a constant temperature magnetic stirring water bath (32°C/5 h). The color of the mixture could be observed from a clear liquid to a milky white cloudy liquid, during the reaction process. After the reaction, the precipitate obtained by centrifugation was washed with ultrapure water and absolute ethanol for many times, and the product quality was improved by differential centrifugation. After completing the above steps, the white powder obtained after drying at 75°C for 6 h was SiO_2 microspheres.

2.4.2 Carboxylation of SiO_2 microspheres

In simpler terms, after sonicating the mixture containing 3.0 mL DMF and 2.0 g maleic anhydride for 15 min, we added 2.0 mL APTES and continued sonication for 15 min until complete dissolution. Next, the mixture and 250 mg of SiO_2 microspheres were added to 20 mL of DMF and mixed at room temperature for 20 h. Finally, the product was obtained by centrifugation and dried in an oven at 35°C for 12 h. The obtained white powders were SiO_2-COOH microspheres.

2.4.3 Synthesis of SiO_2-COOH@MIPs and SiO_2-COOH@NIPs

We shook the acetonitrile solution containing 200 mg SiO_2-COOH, 0.5 mmol template molecule (CAP) and 2.0 mmol functional monomer (MAA) for 2 h and slowly stirred at room temperature for 8 h to form a prepolymerized system. Then, 10 mmol of the cross-linking agent

(EGDMA) and 40.0 mg of the initiator (AIBN) were added to the prepolymerization system, and the mixture was subjected to ultrasonic (15 min), nitrogen (10 min) and water bath heating (60°C/24 h) treatments in sequence. We centrifuged the reacted solution and washed the product with ultrapure water to remove unreacted material, resulting in a white product.

The eluate (methanol/ acetic acid, 9/1, V/V) in which SiO_2-COOH @MIPs was dissolved was sonicated (30 min) and centrifuged (15 min) sequentially to guarantee the elution effect. The whole process was repeated 3-5 times. The final product was dried to obtain SiO_2-COOH@MIPs. For comparison, we prepared non-molecularly imprinted polymers with only difference from SiO_2-COOH@MIPs that SiO_2-COOH@NIPs did not add CAP during the preparation.

2.5 Adsorption studies

CAP solutions of different concentrations were prepared, and their absorbance values were measured at 278 nm using an ultraviolet spectrophotometer. The concentration of CAP solution was taken as the abscissa and the absorbance value as the ordinate to draw the standard curve.

2.5.1 Experiment for adsorption isotherm

We weighed 12 groups of 10 mg SiO_2-COOH@MIPs and SiO_2-COOH@NIPs, and added 5 mL of CAP solution in the concentration range of 0.02-0.13 mg/mL to them, respectively, and shook them at 25℃ for 12 h. After centrifugation, the supernatant was filtered with a sterile microporous membrane of 0.22 μm, and the absorbance value of the supernatant was measured at 278 nm. According to the change of CAP concentration in the solution before and after adsorption, the adsorption capacity Q_e of SiO_2-COOH@MIPs and SiO_2-COOH@NIPs for CAP was

calculated. The calculation formula was as follows[2]:

$$Q_e = \frac{(C_0\text{-}C_e)V}{m} \tag{1}$$

Where Q_e, C_0, C_e, V and m were the equilibrium adsorption capacity (mg/g), initial concentration of CAP solution (mg/mL), concentration of CAP in supernatant (mg/mL), volume of CAP solution (mL), and mass of SiO_2-COOH@MIPs or SiO_2-COOH@NIPs (g), respectively.

2.5.2 Experiment for adsorption kinetic

We weighed 7 groups of 10 mg SiO_2-COOH@MIPs and SiO_2-COOH@NIPs, to which 5 mL of 0.12 mg/mL CAP solution was added, respectively. Shake them for t min (t=10, 20, 30, 40, 50, 60, 70) at a constant temperature of 25°C. After centrifugation, the supernatant was filtered with a sterile microporous membrane of 0.22 μm, and the absorbance value of the supernatant was measured at 278 nm. According to the change of CAP concentration in the solution before and after adsorption, the adsorption capacity Q_t of SiO_2-COOH@MIPs and SiO_2-COOH@NIPs for CAP was calculated. The calculation formula was as follows[2]:

$$Q_t = \frac{(C_0\text{-}C_t)V}{m} \tag{2}$$

Where Q_t (mg/g) was the adsorption capacity at time t, C_t (mg/mL) was the concentration of CAP in the supernatant at time t.

2.5.3 Experiment of selective adsorption

Structural analogs of CAP such as flufenicol (FFC), ofloxacin (OFX), norfloxacin (NOR), and thiamphenicol (TAP) were selected as interference substanced to test the selective adsorption abilities of SiO_2-COOH@MIPs and SiO_2-COOH@NIPs. We weighed 5 groups of 10 mg SiO_2-COOH@MIPs and SiO_2-COOH@NIPs, and added 5 mL of 0.12 mg/mL

CAP solution CAP solution, FFC solution, OFX solution, NOR solution and TAP solution to them, respectively, and shook them for 40 min at 25°C constant temperature. After centrifugation, the supernatant was filtered with 0.22 μm sterile microporous membrane, and the corresponding absorbance values were measured at 278 nm, 266 nm, 294 nm, 285 nm and 226 nm, respectively. The imprinting factor (IF) and selectivity factor (β) of SiO_2-COOH@MIPs and SiO_2-COOH@NIPs were calculated according to the change of the concentration of each substance in the solution before and after adsorption. The calculation formula were as follows[3]:

$$IF=\frac{Q_{SiO_2\text{-}COOH@MIPs}}{Q_{SiO_2\text{-}COOH@NIPs}} \tag{3}$$

$$\beta=\frac{Q_{template}}{Q_{interferent}} \tag{4}$$

Among them, Q_{MIPs} and Q_{NIPs} were the adsorption capacities of SiO_2-COOH@MIPs and SiO_2-COOH@NIPs for various substances; $Q_{template}$ and $Q_{interferent}$ were the adsorption capacities of SiO_2-COOH@MIPs for template molecules and interfering substances.

2.6 The pretreatment of GCE

Add 0.3μm alumina powder and a small amount of ultrapure water to the polishing machine and stir to mix evenly. The glassy carbon electrode was held by fingers and gently placed vertically on the polishing machine to polish to a smooth surface. The polished glassy carbon electrodes were sequentially immersed in ethanol (75%) and ultrapure water and sonicated for 3 min respectively.

2.7 Electrochemical measurements

Electrochemical measurements were performed based on a three-electrode system consisting of a reference electrode, a modified glassy carbon electrode (SiO_2-COOH@MIPs/SnS_2/NiFe-PBA/GCE) and a

counter electrode. The potential range for DPV measurements was -0.2 V to 0.8 V with a pulse period of 0.5 s, a pulse amplitude of 50 mV, and a potential increment of 4 mV. The SiO_2-COOH@MIPs/SnS_2/NiFe-PBA/GCE sensor was submerged in a certain concentration of CAP standard solution or the actual sample extract for 20 min and then naturally dried. Finally, the incubated sensor was inserted into a solution containing 5 mmol/L $[Fe(CN)_6]^{3-/4-}$ and 0.1 mol/L KCl for DPV measurement.

2.8 Preparation of real samples

We tested the performance of the SiO_2-COOH@MIPs/SnS_2/NiFe-PBA/GCE sensor by processing randomly purchased milk samples from the market according to the procedure reported in the literature[4]. Using the standard addition method, the prepared CAP milk samples of different concentrations were placed in a centrifuge at 12,000 rpm for 10 min. Then, the upper fat was removed and the remaining liquid was filtered with a 0.22 μm sterile microporous membrane. Finally, the filtered subnatant was diluted with PBS solution (pH=7.5) for analysis and detection.

3. Results and discussion

3.1.2 SnS_2 nanoflower

The morphology of SnS_2 nanoflowers were characterized by SEM and TEM. The synthesized SnS_2 nanoflowers were provided with a typical three-dimensional flower-like structure with an average petal size of about 1000 nm (Figure S1A and 1B). The main element species and element distribution of SnS_2 nanoflowers were analyzed by EDS. The synthesized SnS_2 nanoflowers mainly contained S and Sn elements through EDS spectroscopy (Figure S1D). In addition, the EDS mapping image (Figure S1C) showed a relatively uniform distribution of S and Sn elements.

Figure S1E showed the image of the XRD data of SnS_2 nanoflowers.

By comparing the main diffraction peaks in the XRD detection results of SnS_2 nanoflowers with the data of SnS_2 (JCPDS No. 23-0677), the synthesis of SnS_2 nanoflowers were relatively successful. Meanwhile, the SnS_2 nanoflowers were further analyzed by XPS. The fully measured spectra of SnS_2 nanoflowers (Figure S1F) further demonstrated the presence of S and Sn elements. In the Figure S1G, two characteristic peaks of S $2p_{3/2}$ at 161.6 eV and S $2p_{1/2}$ at 162.6 eV could be observed, respectively. There were two characteristic peaks of Sn $3d_{5/2}$ at 486.6 eV and Sn $3d_{3/2}$ at 495.1 eV in the spectrum of Sn 3d (Figure S1H)[5]. All the above conclusions indicated that SnS_2 nanoflowers were successfully prepared.

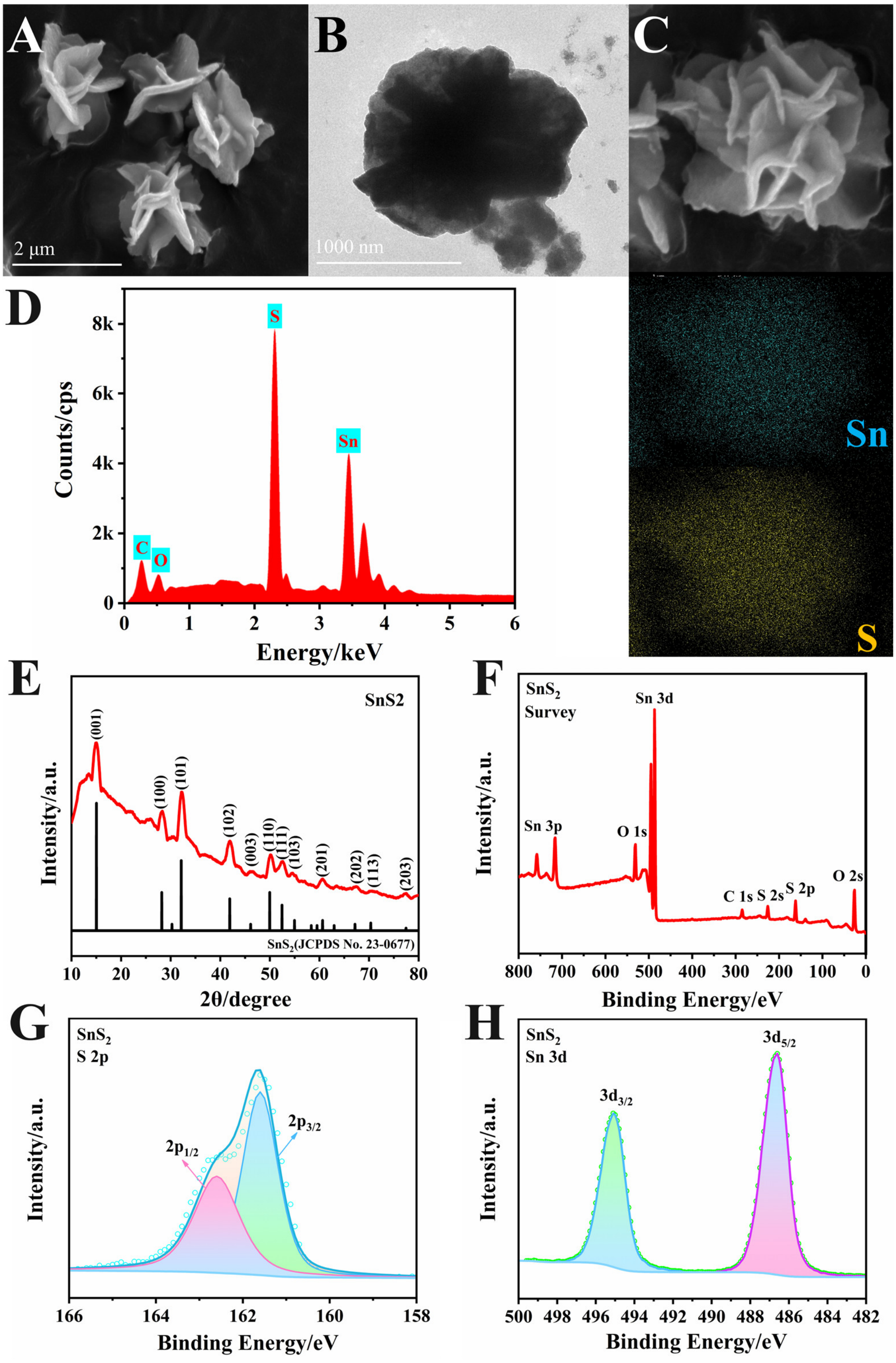

Figure S1. (A) SEM image of NiFe-PBA SnS_2 nanoflowers. (B) TEM image of SnS_2 nanoflowers. (C) EDS mapping image of SnS_2 nanoflowers. (D) EDS spectrum of SnS_2 nanoflowers. (E) XRD image of SnS_2 nanoflowers. XPS spectra of SnS_2 nanoflowers: (F) full-survey spectrum, (G) S 2p spectrum and (H) Sn 3d spectrum.

In order to explore the ability of SnS_2 nanoflowers to increase the specific surface area of the electrode, the Randles-Sevcik equation was used to calculate the electroactive surface area of the bare electrode and the electrode modified with SnS_2 nanoflowers.

First, CV tests of GCE and SnS_2/GCE were performed at different scan rates in 0.1 M KCl solution containing 5 mM $[Fe(CN)_6]^{3-/4-}$. The ECSA of GCE and SnS_2/GCE was then calculated according to the Randles-Sevcik equation. The Randles-Sevcik equation was as follows[6]:

$$Ip = (2.69\times10^5)\, n^{3/2} A D^{1/2} C v^{1/2} \quad (5)$$

where n is the number of electrons produced in the reaction, v is the scan rate (V/s), A is the electroactive surface area of the electrode（cm^2）, and D is the $[Fe(CN)6]^{3-/4-}$ diffusion coefficient (cm^2/s) , C is the $[Fe(CN)_6]^{3-/4-}$ concentration (mol/cm^3), and Ip is the peak current (A).

Figure S2A and 2C were the cyclic voltammetry curves of GCE and SnS_2/GCE, respectively. Figure S2B and 2D were the linear relationship between the square root of the scan rate of GCE and SnS_2/GCE and the oxidation peak current and reduction peak current, respectively. After calculation, the ECSAs of GCE and SnS_2/GCE were 0.073cm^2 and 0.286cm^2, respectively. It could be seen that the ECSA of the electrode with SnS_2 nanoflowers was 3.92 times larger than that of the bare electrode.

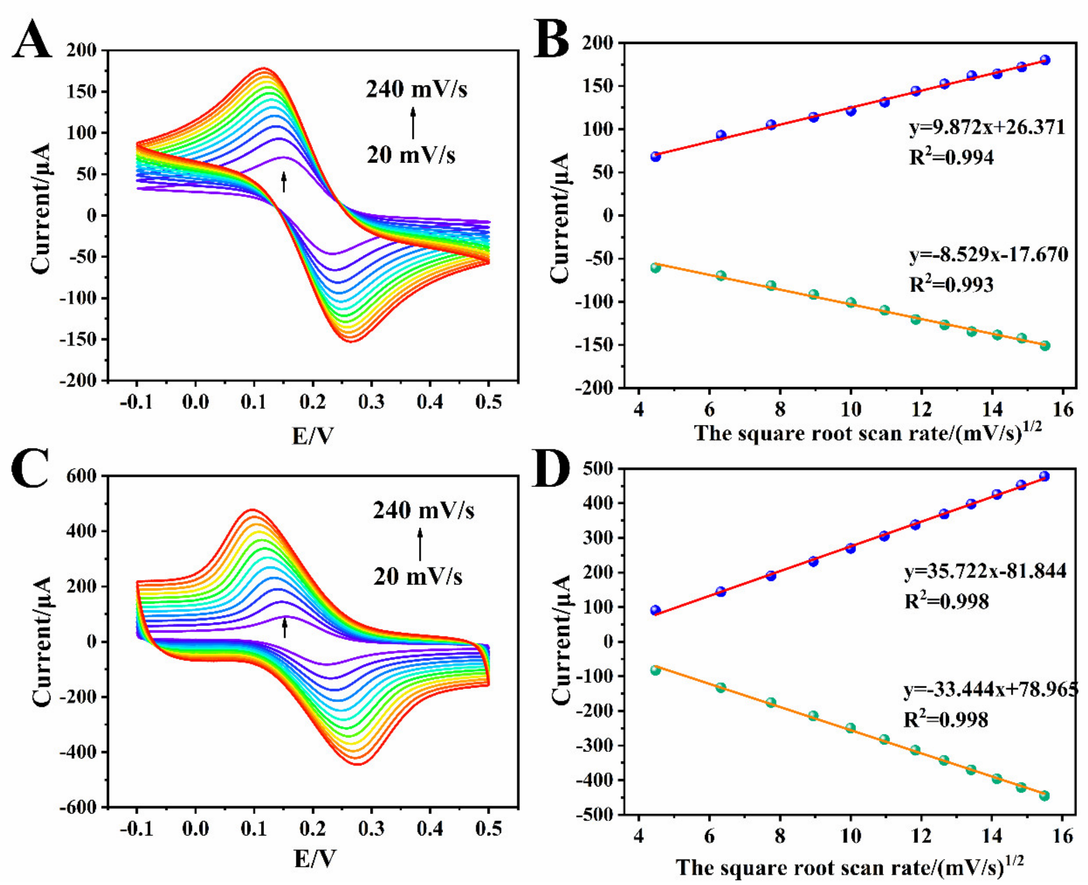

Figure S2. Cyclic voltammetry curves of GCE (A) and SnS_2/GCE (C) at different scan rates. Linear relationship of the square root of scan rate to oxidation peak current and reduction peak current, respectively (GCE(B); SnS_2/GCE (D)).

3.1.3 SiO_2-COOH@MIPs

The morphologies of the synthesized SiO_2 nano-microspheres and SiO_2-COOH@MIPs were characterized by SEM and TEM. The particle size of the synthesized SiO_2 nano-microspheres was relatively uniform, about 200 nm (Figure S3A and 3C). Figure S3B showed that the surface of the SiO_2 nano-microspheres had been successfully wrapped with a layer of MIPs. Meanwhile, the core-shell structure could be more intuitively observed by TEM (Figure S3D), where the thickness of the shell layer was about 80 nm.

We characterized SiO_2, SiO_2-COOH and SiO_2-COOH@MIPs using

FTIR characterizations (Figure S3E). For the spectrum of SiO_2 (curve a), the absorption peaks at 470 cm^{-1} and 801 cm^{-1} came from the bending vibration of Si-O and the symmetric stretching vibration of Si-O, respectively. The antisymmetric stretching vibration of Si-O-Si caused a strong absorption peak at 1110 cm^{-1}, which were characteristic peaks of SiO_2[7]. Curve b showed the spectral image of SiO_2-COOH. The mixed vibration of in-plane bending vibration (C-H) and stretching vibration (C-O) caused the absorption peak at 951 cm^{-1}, the absorption peak at 1645 cm^{-1} was caused by the stretching vibration of C=O in the carboxyl group. The stretching vibration of N-H and O-H caused the absorption peak at 3420 cm^{-1}, indicating that the surface of SiO_2 nano-microspheres has been successfully modified with carboxyl groups[8]. For the spectral image of SiO_2-COOH@MIPs (curve c), the characteristic peaks of COO- and C-O-C at 1157 cm^{-1} and 1254 cm^{-1} were derived from the ester bond of EGDMA. The C=O stretching vibration caused the absorption peak at 1730 cm^{-1} and the -CH stretching vibration of $-CH_2$ and $-CH_3$ caused the absorption peak at 2954 cm^{-1}. These results indicated that MIPs had been successfully synthesized and encapsulated on the SiO_2-COOH surface [9].

Figure S3F revealed the TGA analysis of SiO_2, SiO_2-COOH and SiO_2-COOH@MIPs. The exceptional thermal stability of SiO_2 nano-microspheres was reflected by almost no mass loss of SiO_2 nano-microspheres (curve a). The weight loss of SiO_2-COOH was about 13.47% when the temperature was raised from 60 °C to 380 °C, which could be attributed to the separation of modified carboxyl groups (curve b). For the SiO_2-COOH@MIPs (curve c), there was a significant mass loss (about 81.59%) from 60°C to 420°C due to thermal degradation of the MIPs wrapped on the SiO_2-COOH surface. The above conclusions again proved

that SiO_2, SiO_2-COOH and SiO_2-COOH@MIPs had been successfully prepared.

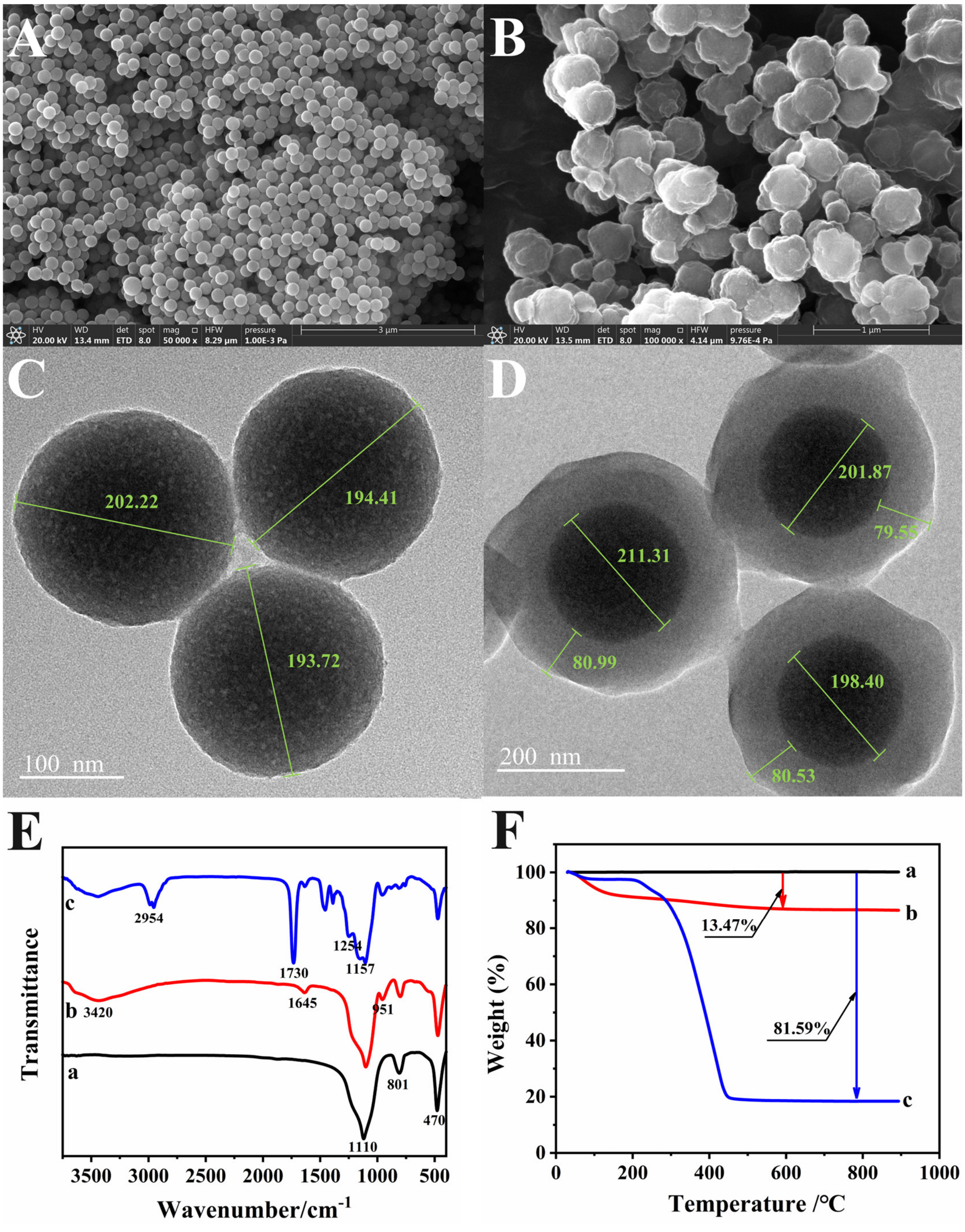

Figure S3. (A) SEM image of SiO_2. (B) SEM image of SiO_2-COOH@MIPs. (C) TEM image of SiO_2. (D) TEM image of SiO_2-COOH@MIPs. (E) FTIR images and (F) TGA images of (a) SiO_2, (b) SiO_2-COOH and (c) SiO_2-COOH@MIPs.

3.4.2 Adsorption kinetics analysis and selective adsorption analysis

The adsorption kinetics curve was an important indicator to study the adsorption capacity of MIPs. The adsorption capacity gradually increased with the increase of time, and the adsorption capacities of SiO_2-COOH@MIPs and SiO_2-COOH@NIPs reached the saturation state when the time reached 40 min (Figure S4A). Compared with SiO_2-COOH@NIPs, SiO_2-COOH@MIPs exhibited better adsorption capacity and faster mass transfer and binding kinetics due to the existence of specific adsorption sites. In order to improve the adsorption efficiency and shorten the reaction time, we could increase the concentration of SiO_2-COOH@MIPs to ensure that CAP was completely adsorbed to achieve quantitative detection of CAP.

The adsorption kinetics analysis was performed by fitting the adsorption data of SiO_2-COOH@MIPs using the pseudo-first-order kinetic equation (Figure S4B) and pseudo-second-order kinetic equation (Figure S4C). The two equations were as follows [10]:

$$ln(Q_e-Q_t)=lnQ_e-k_1t \tag{6}$$

$$\frac{t}{Q_t}=\frac{t}{Q_e}+\frac{1}{k_2 \cdot Q_e^2} \tag{7}$$

Among them, k_1 was a pseudo-first-order rate constant, and k_2 was a pseudo-second-order rate constant. The data obtained by fitting the above two equations were recorded in Table S3.

It could be seen from Table S3 that the two kinetic models had high fitting correlation coefficients (R^2>0.98) for the adsorption process of SiO_2-COOH@MIPs. But the pseudo-second-order kinetic equation had a higher correlation coefficient (R^2=0.996). At the same time, the $Q_{e,cal}$ value (45.745 mg/g) obtained by the pseudo-second-order kinetic equation was

closer to the actual adsorption value (44.300 mg/g) than the $Q_{e,cal}$ value (36.751 mg/g) obtained by the pseudo-first-order kinetic equation. From the above data, it was clear that the adsorption behavior of SiO_2-COOH@MIPs was more in line with the pseudo-second-order kinetic model.

Figure S4D showed the chemical structures of five antibiotics (CAP, FFC, OFX, NOR and TAP). Figure S4E shown the Q_e values of SiO_2-COOH@MIPs and SiO_2-COOH@NIPs for five antibiotics, and the IF and β values calculated from the Q_e values. The results showed that the Q_e value (44.3 mg/g) and the IF value (3.76) of SiO_2-COOH@MIPs for CAP were the largest, which could be attributed to the presence of imprinted cavities on the surface of SiO_2-COOH@MIPs that could specifically recognize CAP, and these cavities could be complementary to the template molecule CAP in shape, size, and spatial distribution. Simultaneously, since the chemical structural formula of TAP was highly similar to CAP, SiO_2-COOH@MIPs also had a high Q_e value for TAP. The IF values of FFC, OFX, NOR and TAP were 1.12, 0.82, 1.43 and 1.50, and the β values were 4.63, 9.06, 8.28 and 3.01, respectively. The above results further demonstrated the high specificity of CAP recognition by SiO_2-COOH@MIPs.

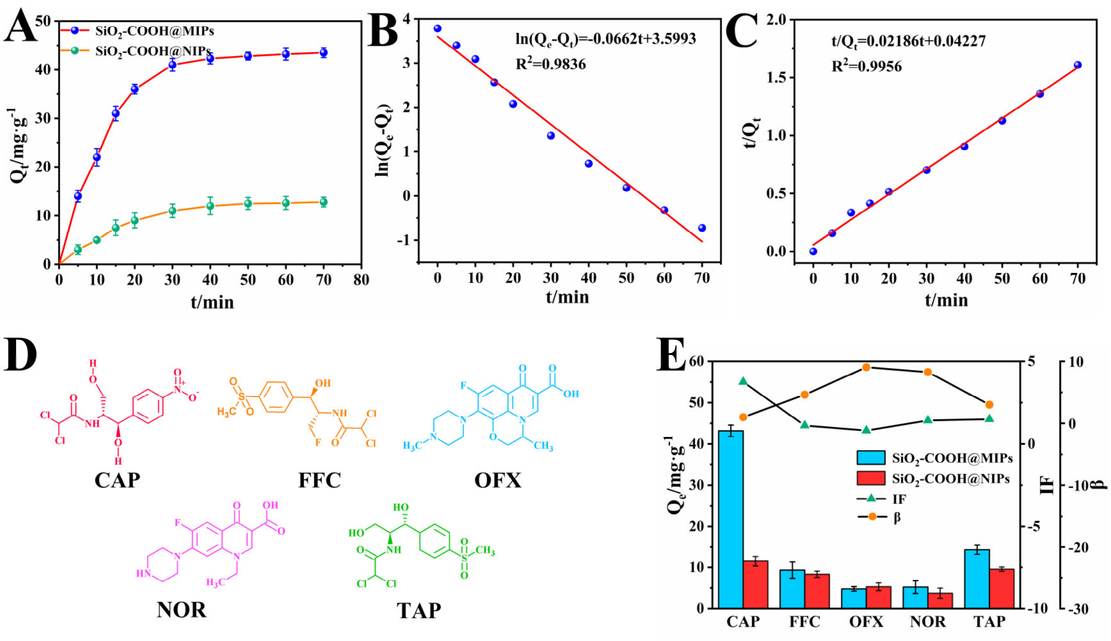

Figure S4. (A) Adsorption kinetics curves for the adsorption of different concentrations of CAP by SiO_2-COOH@MIPs and SiO_2-COOH@NIPs. Pseudo-first-order kinetics equation (B), pseudo-second-order kinetics equation (C) of SiO_2-COOH@MIPs in the adsorption kinetics test of CAP. (D) Chemical structures of five antibiotics CAP, FFC, OFX, NOR and TAP. (E) Adsorption capacity of SiO_2-COOH@MIPs and SiO_2-COOH@NIPs for CAP, FFC, OFX, NOR and TAP.

Table S1

Scatchard parameters of SiO_2-COOH@MIPs and SiO_2-COOH@NIPs for the adsorption of CAP.

Samples	Q_m(mg/g)	K_d(mg/L)	R^2
SiO_2-COOH@MIPs	40.336	1.286	0.9983
	48.761	3.595	0.9955
SiO_2-COOH@NIPs	17.071	64.516	0.9709

Table S2

Langmuir and Freundlich parameters of SiO_2-COOH@MIPs and SiO_2-COOH@NIPs for the adsorption of CAP.

Langmuir			Freundlich		
K_L(L/mg)	Q_m(mg/g)	R^2	K_F(L/mg)	1/n	R^2
0.527	46.208	0.988	12.073	0.366	0.994

Table S3

Pseudo-first-order and Pseudo-second-order parameters of SiO_2-COOH @MIPs for the adsorption of CAP.

Pseudo-first-order			Pseudo-second-order		
k_1	$Q_{e,cal}$ (mg/g)	R^2	k_2	$Q_{e,cal}$(mg/g)	R^2
0.066	36.571	0.984	0.012	45.745	0.996

References

1. Niu, Q.; Bao, C.; Cao, X.; Liu, C.; Wang, H.; Lu, W., Ni-Fe PBA hollow nanocubes as efficient electrode materials for highly sensitive detection of guanine and hydrogen peroxide in human whole saliva. *Biosens Bioelectron* **2019,** 141, 111445.
2. Fan, J.-P.; Xu, X.-K.; Xu, R.; Zhang, X.-H.; Zhu, J.-H., Preparation and characterization of molecular imprinted polymer functionalized with core/shell magnetic particles (Fe_3O_4 @SiO_2@MIP) for the simultaneous recognition and enrichment of four taxoids in Taxus × media. *Chem Eng J.* **2015,** 279, 567-577.
3. Chen, Q.; Liu, X.; Yang, H.; Zhang, S.; Song, H.; Zhu, X., Preparation and evaluation of magnetic graphene oxide molecularly imprinted polymers (MIPs-GO-Fe_3O_4@SiO_2) for the analysis and separation of tripterine. *React Funct Polym.* **2021,** 169, 105055.
4. Yue, F.; Li, H.; Kong, Q.; Liu, J.; Wang, G.; Li, F.; Yang, Q.; Chen, W.; Guo, Y.; Sun, X., Selection of broad-spectrum aptamer and its application in fabrication of aptasensor for detection of aminoglycoside antibiotics residues in milk. *Sensor Actuat B-Chem.* **2022,** 351, 130959.
5. Zhu, Q.; Gu, D.; Liu, Z.; Huang, B.; Li, X., Au-modified 3D SnS_2 nano-flowers for low-temperature NO2 sensors. *Sensor Actuat B-Chem.* **2021,** 349, 130775.
6. Geng, L.; Huang, J.; Zhai, H.; Shen, Z.; Han, J.; Yu, Y.; Fang, H.; Li, F.; Sun, X.; Guo, Y., Molecularly imprinted electrochemical sensor based on multi-walled carbon nanotubes for specific recognition and determination of chloramphenicol in milk. *Microchem J.* **2022,** 182, 107887.

7. Xu, Y.; Huang, T.; Wang, S.; Meng, M.; Yan, Y., SiO_2-coated molecularly imprinted sensor based on Si quantum dots for selective detection of catechol in river water. *J Environ Chem Eng* **2022,** 10, 106850.

8. Liu, Y.; Huang, Z.; He, W.; Chen, M.; Tu, W.; Zhu, M.; Gan, D.; Liu, S., Multifunctional stable PDA/RGO/MOFs&SiO_2-COOH membrane with excellent flux and anti-fouling performance for the separation of organic dye and oil/water. *Surf Interfaces.* **2022,** 33, 102183.

9. Sun, J.; Guo, W.; Ji, J.; Li, Z.; Yuan, X.; Pi, F.; Zhang, Y.; Sun, X., Removal of patulin in apple juice based on novel magnetic molecularly imprinted adsorbent Fe_3O_4@SiO_2@CS-GO@MIP. *Lwt* **2020,** 118, 108854.

10. Hao, Y.; Gao, Y.; Song, H.; Niu, Y.; Chen, X.; Liu, X.; Gao, R.; Wang, S., Fabrication of metal coordination-synergistic magnetic imprinted microspheres based on ligand-free Fe_3O_4-Cu for specific recognition of bovine hemoglobin. *Talanta* **2021,** 233, 122496.
